# Crystallographic coincidence of two bridging species in a dinuclear Co^III^ ethynyl­benzene complex

**DOI:** 10.1107/S1600536811019969

**Published:** 2011-06-04

**Authors:** Wesley A. Hoffert, Matthew P. Shores

**Affiliations:** a Department of Chemistry, Colorado State University, Fort Collins, CO 80523-1872, USA

## Abstract

In the title compound, *trans*,*trans*-[μ-(*m*-phenyl­ene)bis­(ethyne-1,2-di­yl)]bis­[chlorido(1,4,8,11-tetra­aza­cyclo­tetra­deca­ne)cobalt(III)]–*trans*,*trans*-[μ-(5-bromo-*m*-phenyl­ene)bis­(ethyne-1,2-di­yl)]bis­[chlorido(1,4,8,11-tetra­aza­cyclo­tetra­deca­ne)cobalt(III)]–tetra­phenyl­borate–acetone (0.88/0.12/2/4), [Co_2_(C_12_H_4_)Cl_2_(C_10_H_24_N_4_)_2_]_0.88_[Co_2_(C_10_H_3_Br)Cl_2_(C_10_H_24_N_4_)_2_]_0.12_(C_24_H_20_B)_2_·4C_3_H_6_O, with the exception of the acetyl­ene and bromine groups, all atomic postitions are the same in the two compounds and are modeled at full occupancy. The Co^III^ ions are six-coordinate with acetyl­ide and chloride ligands bound to the axial sites and the N atoms from the cyclam rings coordinated at the equatorial positions. N—H⋯O and N—H⋯Cl hydrogen-bonding interactions help to consolidate the crystal packing.

## Related literature

Metallodendrimers are of inter­est for their unique catalytic and optical properties, see: Mery & Astruc (2006 ▶); Onitsuka & Takahashi (2003 ▶). For Pt(II)- and Ru(II)-containing dendrimers based on a 1,3,5-triethynyl­benzene (H_3_TEB) linkage, see: Onitsuka *et al.* (2004 ▶); McDonagh *et al.* (2003 ▶). For a discussion of the structural similarity between halogen and ethynyl substituents, see: Robinson *et al.* (1998 ▶). For related metal–acetyl­ide structures, see: Weyland *et al.* (1998 ▶); Onitsuka *et al.* (2004 ▶). For the structure of [(cyclam)CoCl_2_]Cl, see: Ivaniková *et al.* (2006 ▶). For the preparation of *trans*-[(cyclam)CoCl_2_]Cl, see: Bosnich *et al.* (1965 ▶). General Sonogashira conditions were used to prepare a mixture of 1,3,5-triethynyl­benzene and 1-bromo-3,5-diethynyl­benzene (Weber *et al.*, 1988 ▶).
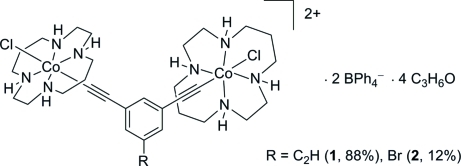


## Experimental

### 

#### Crystal data

[Co_2_(C_12_H_4_)Cl_2_(C_10_H_24_N_4_)_2_]_0.88_[Co_2_(C_10_H_3_Br)Cl_2_(C_10_H_24_N_4_)_2_]_0.12_·(C_24_H_20_B)_2_·4C_3_H_6_O
*M* *_r_* = 1614.61
Triclinic, 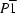

*a* = 10.1434 (4) Å
*b* = 17.1412 (7) Å
*c* = 25.5250 (11) Å
α = 92.609 (1)°
β = 96.864 (1)°
γ = 104.323 (1)°
*V* = 4256.2 (3) Å^3^
*Z* = 2
Mo *K*α radiation
μ = 0.56 mm^−1^
*T* = 120 K
0.60 × 0.30 × 0.30 mm

#### Data collection

Bruker APEXII CCD area-detector diffractometer
Absorption correction: multi-scan (*SADABS*; Bruker, 2009 ▶) *T*_min_ = 0.729, *T*_max_ = 0.850
40567 measured reflections
25801 independent reflections
20379 reflections with *I* > 2σ(*I*)
*R*_int_ = 0.016

#### Refinement

*R*[*F* ^2^ > 2σ(*F* ^2^)] = 0.037
*wR*(*F* ^2^) = 0.097
*S* = 1.02
25801 reflections
1001 parameters
H-atom parameters constrained
Δρ_max_ = 0.52 e Å^−3^
Δρ_min_ = −0.42 e Å^−3^

### 

Data collection: *APEX2* (Bruker, 2009 ▶); cell refinement: *SAINT* (Bruker, 2009 ▶); data reduction: *SAINT*; program(s) used to solve structure: *SHELXTL* (Sheldrick, 2008 ▶); program(s) used to refine structure: *SHELXTL*; molecular graphics: *SHELXTL*; software used to prepare material for publication: *SHELXTL*.

## Supplementary Material

Crystal structure: contains datablock(s) I, global. DOI: 10.1107/S1600536811019969/qm2008sup1.cif

Structure factors: contains datablock(s) I. DOI: 10.1107/S1600536811019969/qm2008Isup2.hkl

Supplementary material file. DOI: 10.1107/S1600536811019969/qm2008Isup3.mol

Additional supplementary materials:  crystallographic information; 3D view; checkCIF report

**Fig. 1.**
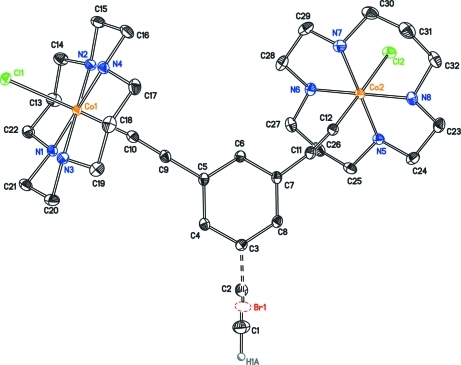
Structure of the superimposed complex cations present in 1 and 2 with atomic numbering scheme and thermal ellipsoids rendered at 40° probability. Orange, green, blue, gray, and red ellipsoids represent cobalt, chlorine, nitrogen, carbon, and bromine atoms respectively. With the exception of the acetylenic hydrogen (H1A, represented by a gray shaded sphere), hydrogen atoms have been omitted for clarity.

Enhanced figure: interactive version of Fig. 1

## Figures and Tables

**Table 1:** Hydrogen-bond geometry (Å, °)

*D*—H⋯*A*	*D*—H	H⋯*A*	*D*⋯*A*	*D*—H⋯*A*
N5—H5⋯Cl2^i^	0.93	2.48	3.2377 (12)	139
N6—H6⋯O3	0.93	2.15	2.9440 (19)	143
N7—H7⋯O2	0.93	2.11	2.9894 (17)	157
N8—H8⋯O1	0.93	2.03	2.8730 (17)	149

Symmetry code: (i) 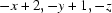.

**Fig. 2.**
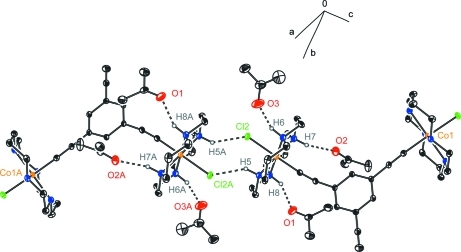
Hydrogen bonding interactions present in the solid state structures of 1 and 2. Thermal ellipsoids are rendered at 40% probability. Red ellipsoids represent oxygen atoms. Otherwise, the color scheme is identical to that found in Figure 1. Tetraphenylborate anions, the acetone molecule that includes O4 (which does not participate in H-bonding), the bromine substituent present in 2, and hydrogen atoms that do not participate in H-bonding have been omitted.

## supplementary crystallographic information

### Comment

From a technological standpoint, metallodendrimers are of interest for their unique catalytic and optical properties (Mery & Astruc, 2006; Onitsuka & Takahashi, 2003). A particular subset of metallodendrimers based on ethynylbenzene have been pursued because of their structural rigidity and topological anisotropy. Although a variety of Pt(II)- and Ru(II)-containing dendrimers based on a 1,3,5-triethynylbenzene (H_3_TEB) linkage have been reported (Onitsuka *et al.*, 2004; McDonagh *et al.*, 2003), we are interested in the properties of first row transition metal TEB complexes for potential applications in molecular magnetism (Weyland *et al.*, 1998). For elaboration to higher nuclearity species, the inclusion of an axially coordinated anionic ligand that is poized for substitution is vital.

The synthesis of these macromolecules can be accomplished by divergent or convergent pathways; regardlesss, each strategy hinges upon the isolation of structurally characterized "building blocks" prior to dendrimer assembly. The preparation of complexes that contain first-row metals is synthetically challenging because of their high kinetic lability relative to their second and third row counterparts. In that respect, our initial synthetic targets contain Co^III^ because of its relative inertness.

The combination of H_3_TEB with two equivalents of *trans*-[(cyclam)CoCl_2_]Cl produces the dinuclear Co^III^ arylacetylide-bridged complex 1 in good yield (Figure 1). Initial refinement attempts on high quality X-ray data did not converge satisfactorily, as the third aromatic substituent showed apparent disorder of the alkynyl group. However, structure refinement proceeds smoothly if compositional disorder is invoked. The accepted method for the preparation of 1,3,5-triethynylbenzene involves Sonogashira coupling between 1,3,5-tribromobenzene and trimethylsilylacetylene (Weber *et al.*, 1988). On one occasion, following the protocol resulted in a batch of TEB containing a sizeable amount of 1-bromo-3,5-diethynylbenzene (Robinson *et al.*, 1998), indicating incomplete substitution. The impurity was carried through several purification steps, eventually affording a mixture of the ethynyl- (1) and bromo- (2) substituted complexes. The crystal structure revealed that both components of the ligand mixture were incorporated into metal complexes and the atomic sites were superimposed. During structure refinement, the compositional disorder at the aromatic 1 position was modeled with a free variable. Final site occupancy factors indicate that the two ligand components are present in an 88:12 1:2 ratio. This compares favorably with subsequent ^1^H NMR analysis of the batch of "H_3_TEB" ligand, which shows resonance integrations in an 87:13 H_3_TEB:H_2_BrTEB ratio.

The molecular structure of the complex cations in 1 and 2 are shown in Figure 1. Each pseudo-octahedral Co^III^ center coordinates four nitrogen atoms from the cyclam rings at the equitorial positions with an average Co—N bond length of 1.9767 (11) Å, which is only slightly longer than the corresponding bond length from the reported structure of *trans*-[(cyclam)CoCl_2_]Cl (1.9741 (12); Ivaniková *et al.*, 2006). Anionic chloride and acetylide ligands occupy the axial Co^III^ coordination sites with average metal-ligand distances of 2.3076 (4) and 1.8770 (14) respectively. The former bond length is significantly longer than the average Co—Cl distance in *trans*-[(cyclam)CoCl_2_]Cl, suggesting that the arylacetylide ligand imparts a stronger *trans* influence than chloride. The cationic charge is balanced by the presence of two tetraphenylborate anions, and the asymmetric unit includes four molecules of acetone.

Shown in Figure 2, the crystal packing in 1 and 2 is influenced by several weak hydrogen bonding interactions. Notably, the complex cations experience a dimeric interaction through pairwise N—H···Cl contacts with a complex in a neighboring unit cell. Furthermore, three of the four acetone molecules participate in hydrogen bonds through the cyclam N–H groups.

In summary, a mixture of H_3_TEB and 1-bromo-3,5-diethynylbenzene combined with *trans*-[(cyclam)CoCl_2_]Cl to yield a co-crystallized mixture of 1 and 2. The compounds are superimposed in the solid state with the exception of the 5-position acetylene and bromine groups. Using a free variable to model the compositional disorder, we conclude that the two compounds are present in a 88:12 ratio. The first coordination sphere for each Co^III^ ion includes an axially replaceable chloride ligand, which is a necessary condition for future metallodendrimer assembly. This result exemplifies the key role of crystallographic analysis in organometallic synthesis development.

### Experimental

*trans*-[(cyclam)CoCl_2_]Cl was prepared by a previously descibed method (Bosnich *et al.* 1965). General Sonogashira conditions were used to prepare a mixture of 1,3,5-triethynylbenzene and 1-bromo-3,5-diethynylbenzene (Weber *et al.*, 1988). Triethylamine was purchased from Sigma-Aldrich and was distilled prior to use.

Elemental analysis was performed by Robertson Microlit in Madison, NJ.

Preparation of 1 and 2: Triethylamine (0.34 ml, 2.42 mmol) was added to a 100 ml round-bottomed flask containing a green methanolic (10 ml) solution of [(cyclam)CoCl_2_]Cl (233 mg, 0.637 mmol) and freshly sublimed mixture (45.5 mg) of 1,3,5-triethynylbenzene (87% by ^1^H NMR) and 1-bromo-3,5-diethynylbenzene (13% by ^1^H NMR). The flask was fitted with a condenser tube and the solution was refluxed for 24 h, during which time the solution turned orange-brown. The solvent was removed by rotary evaporation, and the resulting red-brown residue was washed with 10 ml of absolute ethanol, causing an orange solid to precipitate. The solid was isolated by filtration, washed with ethanol (3 × 3 ml) and diethyl ether (3 × 3 ml) and dried in air to afford 92.1 mg of an orange solid. The orange solid was dissolved in methanol (10 ml) and a solution of excess sodium tetraphenylborate in methanol (5 ml) was added, causing a salmon-colored solid to precipitate. The solid was isolated by filtration, washed with methanol (3 × 3 ml) and diethyl ether (3 × 3 ml) and dried in air to afford 131 mg of product (0.094 mmol, 30% based on [(cyclam)CoCl_2_]Cl). Anal. Calcd. for C_85.74_H_103.87_B_2_Br_0.13_Cl_2_Co_2_N_8_O_2_: C, 68.68; H, 6.98; N, 7.47. Found: C, 68.33; H, 7.02; N, 7.85. Single crystals suitable for X-ray analysis were grown by diffusing diethyl ether vapor into a concentrated solution of the compound in acetone for 2 days.

### Refinement

Displacement parameters for all non-hydrogen atoms were refined anisotropically. Hydrogen atoms were assigned to ideal positions and were refined using a riding model where the displacement parameters were set at 1.2 times those of the attached carbon or nitrogen atoms (1.5 times for methyl protons).

### Crystal data

**Table:**

[Co_2_(C_12_H_4_)Cl_2_(C_10_H_24_N_4_)_2_]_0.88_[Co_2_(C_10_H_3_Br)Cl_2_(C_10_H_24_N_4_)_2_]_0.12_·(C_24_H_20_B)_2_·4C_3_H_6_O	*Z* = 2
*M**_r_* = 1614.61	*F*(000) = 1711.8
Triclinic, *P*1	*D*_x_ = 1.260 Mg m^−^^3^
Hall symbol: -P 1	Mo *K*α radiation, λ = 0.71073 Å
*a* = 10.1434 (4) Å	Cell parameters from 9660 reflections
*b* = 17.1412 (7) Å	θ = 2.1–33.1°
*c* = 25.5250 (11) Å	µ = 0.56 mm^−^^1^
α = 92.609 (1)°	*T* = 120 K
β = 96.864 (1)°	Block, orange
γ = 104.323 (1)°	0.60 × 0.30 × 0.30 mm
*V* = 4256.2 (3) Å^3^	

### Data collection

**Table:**

Bruker APEXII CCD area-detector diffractometer	25801 independent reflections
Radiation source: fine-focus sealed tube	20379 reflections with *I* > 2σ(*I*)
graphite	*R*_int_ = 0.016
φ and ω scans	θ_max_ = 30.5°, θ_min_ = 2.0°
Absorption correction: multi-scan (*SADABS*; Bruker, 2009)	*h* = −14→14
*T*_min_ = 0.729, *T*_max_ = 0.850	*k* = −24→23
40567 measured reflections	*l* = −36→35

### Refinement

**Table:**

Refinement on *F*^2^	Primary atom site location: structure-invariant direct methods
Least-squares matrix: full	Secondary atom site location: difference Fourier map
*R*[*F*^2^ > 2σ(*F*^2^)] = 0.037	Hydrogen site location: inferred from neighbouring sites
*wR*(*F*^2^) = 0.097	H-atom parameters constrained
*S* = 1.02	*w* = 1/[σ^2^(*F*_o_^2^) + (0.0448*P*)^2^ + 1.6289*P*] where *P* = (*F*_o_^2^ + 2*F*_c_^2^)/3
25801 reflections	(Δ/σ)_max_ = 0.002
1001 parameters	Δρ_max_ = 0.52 e Å^−^^3^
0 restraints	Δρ_min_ = −0.42 e Å^−^^3^

### Special details

**Table:**

Experimental. Although we cannot explain the source of the Hirshfield tests that give rise to the B– and C-level alerts, there is no evidence of substitutional disorder at the atomic sites mentioned in the alerts. The reason for the presence of a non-integer number of atoms is due to substitutional disorder between bromine and acetylene substituents as descibed in the text. Four reflections were omitted from refinement due to beamstop interference. Probable reasons for the missing cusp of data include beamstop interference and data truncation at resolutions higher than 0.70 Å during the initial stages of refinement. The low "solvent" *U*_eq_ in C88 C91 (the central C atoms in two of the acetone molecules) compared to neighboring atoms cannot be explained by substitutional disorder or incorrect atom type. However, we note that the differences in *U*_eq_ are relatively minor. The four D—H groups on the cyclam rings do not interact with acceptors. This has been checked and the exception is apparently common for N—H groups. One of the tetraphenylborate anions and one of the acetone molecules do not have their centers of gravity within the unit cell. Since neither molecule is the main species, there is no cause for alarm. The s.u. values for the unit cell angles have been checked, and the fact that all angles have the same s.u. is purely coincedental. The long C(sp2)-C(sp1) bonds noted for C5—C9 and C7—C11 appear to be real. Since these bonds include an aromatic carbon, this may be a false alarm.
Geometry. All e.s.d.'s (except the e.s.d. in the dihedral angle between two l.s. planes) are estimated using the full covariance matrix. The cell e.s.d.'s are taken into account individually in the estimation of e.s.d.'s in distances, angles and torsion angles; correlations between e.s.d.'s in cell parameters are only used when they are defined by crystal symmetry. An approximate (isotropic) treatment of cell e.s.d.'s is used for estimating e.s.d.'s involving l.s. planes.
Refinement. Refinement of *F*^2^ against ALL reflections. The weighted *R*-factor *wR* and goodness of fit *S* are based on *F*^2^, conventional *R*-factors *R* are based on *F*, with *F* set to zero for negative *F*^2^. The threshold expression of *F*^2^ > σ(*F*^2^) is used only for calculating *R*-factors(gt) *etc*. and is not relevant to the choice of reflections for refinement. *R*-factors based on *F*^2^ are statistically about twice as large as those based on *F*, and *R*- factors based on ALL data will be even larger.

### Fractional atomic coordinates and isotropic or equivalent isotropic displacement parameters (Å^2^)

**Table:**

	*x*	*y*	*z*	*U*_iso_*/*U*_eq_	Occ. (<1)
Co1	0.569900 (17)	0.158047 (10)	0.401709 (7)	0.01310 (4)	
Co2	0.839505 (18)	0.470946 (10)	0.098236 (7)	0.01540 (4)	
Cl1	0.42722 (3)	0.056323 (18)	0.439190 (13)	0.01862 (6)	
Cl2	0.78675 (3)	0.45872 (2)	0.006915 (13)	0.02340 (7)	
N1	0.72417 (11)	0.10677 (6)	0.41499 (4)	0.0158 (2)	
H1	0.7961	0.1377	0.3995	0.019*	
N2	0.50440 (11)	0.10322 (7)	0.33001 (4)	0.0173 (2)	
H2	0.5574	0.1342	0.3074	0.021*	
N3	0.63209 (11)	0.21325 (6)	0.47314 (4)	0.0163 (2)	
H3	0.5739	0.1843	0.4950	0.020*	
N4	0.41663 (11)	0.20999 (7)	0.38934 (4)	0.0170 (2)	
H4	0.3477	0.1818	0.4074	0.020*	
N5	1.03433 (12)	0.51527 (7)	0.09070 (5)	0.0187 (2)	
H5	1.0394	0.5099	0.0546	0.022*	
N6	0.85298 (12)	0.35773 (7)	0.09466 (5)	0.0208 (2)	
H6	0.8449	0.3419	0.0588	0.025*	
N7	0.64379 (12)	0.42663 (7)	0.10699 (5)	0.0215 (2)	
H7	0.6393	0.4324	0.1431	0.026*	
N8	0.82626 (12)	0.58398 (7)	0.10179 (5)	0.0202 (2)	
H8	0.8332	0.5997	0.1376	0.024*	
B1	1.27714 (15)	0.10990 (8)	0.14789 (6)	0.0172 (3)	
B2	0.21036 (15)	0.23378 (8)	0.57547 (6)	0.0156 (3)	
O1	0.87863 (16)	0.68935 (8)	0.19688 (5)	0.0460 (3)	
O2	0.54710 (14)	0.43689 (8)	0.21263 (5)	0.0436 (3)	
O3	0.86420 (18)	0.24635 (10)	0.00500 (7)	0.0645 (5)	
O4	1.3523 (2)	0.69047 (12)	0.18809 (10)	0.1043 (8)	
C1	1.3457 (4)	0.50591 (15)	0.40696 (12)	0.0352 (6)	0.8771 (17)
H1A	1.4333	0.5262	0.4274	0.042*	0.8771 (17)
C2	1.2357 (3)	0.48043 (18)	0.38132 (13)	0.0226 (6)	0.8771 (17)
C3	1.10471 (13)	0.44906 (7)	0.35041 (5)	0.0168 (2)	
C4	1.00746 (13)	0.38761 (7)	0.36879 (5)	0.0166 (2)	
H4A	1.0280	0.3676	0.4021	0.020*	
C5	0.87944 (13)	0.35534 (7)	0.33824 (5)	0.0156 (2)	
C6	0.85153 (13)	0.38488 (7)	0.28917 (5)	0.0162 (2)	
H6A	0.7653	0.3627	0.2681	0.019*	
C7	0.94862 (13)	0.44665 (7)	0.27051 (5)	0.0156 (2)	
C8	1.07510 (13)	0.47938 (7)	0.30170 (5)	0.0169 (2)	
H8A	1.1409	0.5222	0.2898	0.020*	
C9	0.77736 (14)	0.29242 (8)	0.35681 (5)	0.0176 (2)	
C10	0.69030 (13)	0.24154 (8)	0.37286 (5)	0.0174 (2)	
C11	0.91742 (14)	0.47036 (8)	0.21806 (5)	0.0188 (3)	
C12	0.88563 (13)	0.47697 (8)	0.17195 (5)	0.0181 (2)	
C13	0.65879 (16)	0.01084 (9)	0.33459 (6)	0.0256 (3)	
H13A	0.6640	−0.0434	0.3214	0.031*	
H13B	0.7231	0.0513	0.3171	0.031*	
C14	0.51399 (15)	0.01905 (8)	0.31905 (6)	0.0237 (3)	
H14A	0.4855	0.0027	0.2808	0.028*	
H14B	0.4503	−0.0177	0.3390	0.028*	
C15	0.36217 (14)	0.11045 (9)	0.31500 (6)	0.0217 (3)	
H15A	0.2968	0.0710	0.3328	0.026*	
H15B	0.3354	0.1000	0.2762	0.026*	
C16	0.36136 (15)	0.19552 (9)	0.33246 (6)	0.0223 (3)	
H16A	0.4190	0.2345	0.3118	0.027*	
H16B	0.2666	0.2022	0.3268	0.027*	
C17	0.44139 (14)	0.29657 (8)	0.40764 (6)	0.0209 (3)	
H17A	0.3556	0.3139	0.3992	0.025*	
H17B	0.5119	0.3294	0.3884	0.025*	
C18	0.48881 (14)	0.31183 (8)	0.46657 (6)	0.0214 (3)	
H18A	0.4215	0.2755	0.4854	0.026*	
H18B	0.4899	0.3681	0.4777	0.026*	
C19	0.63025 (14)	0.29889 (8)	0.48330 (6)	0.0212 (3)	
H19A	0.6977	0.3335	0.4636	0.025*	
H19B	0.6581	0.3153	0.5215	0.025*	
C20	0.77018 (14)	0.20181 (8)	0.49135 (6)	0.0209 (3)	
H20A	0.7900	0.2099	0.5304	0.025*	
H20B	0.8415	0.2413	0.4762	0.025*	
C21	0.76967 (14)	0.11677 (8)	0.47297 (5)	0.0195 (3)	
H21A	0.8628	0.1082	0.4807	0.023*	
H21B	0.7059	0.0772	0.4912	0.023*	
C22	0.70448 (14)	0.02212 (8)	0.39365 (6)	0.0208 (3)	
H22A	0.6348	−0.0139	0.4117	0.025*	
H22B	0.7919	0.0063	0.4014	0.025*	
C23	0.95084 (16)	0.63438 (9)	0.08301 (6)	0.0252 (3)	
H23A	0.9649	0.6918	0.0954	0.030*	
H23B	0.9407	0.6298	0.0439	0.030*	
C24	1.07054 (15)	0.60369 (8)	0.10543 (6)	0.0243 (3)	
H24A	1.1543	0.6309	0.0906	0.029*	
H24B	1.0879	0.6144	0.1444	0.029*	
C25	1.13921 (14)	0.47734 (9)	0.11654 (6)	0.0240 (3)	
H25A	1.1435	0.4848	0.1555	0.029*	
H25B	1.2304	0.5044	0.1071	0.029*	
C26	1.10616 (16)	0.38797 (10)	0.09968 (6)	0.0276 (3)	
H26A	1.0930	0.3809	0.0605	0.033*	
H26B	1.1857	0.3671	0.1127	0.033*	
C27	0.97936 (16)	0.33818 (9)	0.11963 (6)	0.0259 (3)	
H27A	0.9723	0.2801	0.1118	0.031*	
H27B	0.9880	0.3487	0.1585	0.031*	
C28	0.72867 (16)	0.30731 (9)	0.11401 (7)	0.0278 (3)	
H28A	0.7401	0.3118	0.1532	0.033*	
H28B	0.7140	0.2499	0.1015	0.033*	
C29	0.60835 (16)	0.33832 (9)	0.09228 (7)	0.0279 (3)	
H29A	0.5899	0.3277	0.0533	0.034*	
H29B	0.5252	0.3109	0.1074	0.034*	
C30	0.53936 (15)	0.46438 (10)	0.08077 (7)	0.0294 (3)	
H30A	0.4482	0.4378	0.0904	0.035*	
H30B	0.5348	0.4559	0.0419	0.035*	
C31	0.57201 (17)	0.55400 (11)	0.09648 (7)	0.0325 (4)	
H31A	0.5845	0.5623	0.1356	0.039*	
H31B	0.4927	0.5746	0.0827	0.039*	
C32	0.69956 (16)	0.60282 (10)	0.07628 (7)	0.0291 (3)	
H32A	0.6911	0.5912	0.0375	0.035*	
H32B	0.7067	0.6611	0.0834	0.035*	
C33	1.16400 (14)	0.02199 (8)	0.13641 (6)	0.0193 (3)	
C34	1.15249 (15)	−0.03715 (8)	0.17315 (6)	0.0235 (3)	
H34	1.2184	−0.0278	0.2040	0.028*	
C35	1.04886 (17)	−0.10891 (9)	0.16644 (7)	0.0295 (3)	
H35	1.0451	−0.1471	0.1924	0.035*	
C36	0.95161 (17)	−0.12430 (9)	0.12182 (8)	0.0351 (4)	
H36	0.8801	−0.1728	0.1170	0.042*	
C37	0.95980 (18)	−0.06811 (10)	0.08420 (8)	0.0368 (4)	
H37	0.8941	−0.0782	0.0533	0.044*	
C38	1.06453 (16)	0.00323 (9)	0.09168 (7)	0.0277 (3)	
H38	1.0684	0.0407	0.0652	0.033*	
C39	1.21641 (13)	0.16870 (8)	0.18652 (5)	0.0183 (2)	
C40	1.10744 (14)	0.13930 (8)	0.21550 (6)	0.0214 (3)	
H40	1.0669	0.0828	0.2130	0.026*	
C41	1.05613 (15)	0.18934 (9)	0.24777 (6)	0.0252 (3)	
H41	0.9828	0.1665	0.2668	0.030*	
C42	1.11142 (15)	0.27216 (9)	0.25222 (6)	0.0261 (3)	
H42	1.0766	0.3065	0.2740	0.031*	
C43	1.21814 (16)	0.30364 (9)	0.22429 (6)	0.0258 (3)	
H43	1.2568	0.3604	0.2267	0.031*	
C44	1.26997 (15)	0.25332 (8)	0.19253 (6)	0.0221 (3)	
H44	1.3443	0.2769	0.1742	0.027*	
C45	1.42846 (14)	0.09960 (8)	0.17397 (5)	0.0181 (2)	
C46	1.52986 (14)	0.16203 (8)	0.20381 (6)	0.0206 (3)	
H46	1.5058	0.2097	0.2150	0.025*	
C47	1.66472 (15)	0.15672 (9)	0.21771 (6)	0.0264 (3)	
H47	1.7301	0.2003	0.2380	0.032*	
C48	1.70339 (16)	0.08796 (10)	0.20199 (7)	0.0312 (3)	
H48	1.7954	0.0845	0.2107	0.037*	
C49	1.60600 (16)	0.02451 (10)	0.17341 (7)	0.0311 (3)	
H49	1.6307	−0.0232	0.1628	0.037*	
C50	1.47178 (15)	0.03053 (8)	0.16026 (6)	0.0245 (3)	
H50	1.4064	−0.0142	0.1411	0.029*	
C51	1.30157 (14)	0.15000 (7)	0.09106 (5)	0.0183 (2)	
C52	1.22093 (15)	0.19872 (8)	0.06825 (6)	0.0237 (3)	
H52	1.1524	0.2111	0.0871	0.028*	
C53	1.23718 (17)	0.22961 (9)	0.01927 (6)	0.0293 (3)	
H53	1.1803	0.2624	0.0054	0.035*	
C54	1.33574 (17)	0.21279 (9)	−0.00925 (6)	0.0290 (3)	
H54	1.3480	0.2342	−0.0425	0.035*	
C55	1.41599 (17)	0.16437 (10)	0.01146 (6)	0.0302 (3)	
H55	1.4832	0.1517	−0.0079	0.036*	
C56	1.39934 (15)	0.13410 (9)	0.06036 (6)	0.0249 (3)	
H56	1.4565	0.1012	0.0737	0.030*	
C57	0.12531 (13)	0.17868 (7)	0.61823 (5)	0.0161 (2)	
C58	0.18508 (15)	0.16756 (9)	0.66888 (6)	0.0217 (3)	
H58	0.2798	0.1926	0.6794	0.026*	
C59	0.11139 (17)	0.12135 (9)	0.70445 (6)	0.0276 (3)	
H59	0.1564	0.1156	0.7384	0.033*	
C60	−0.02683 (16)	0.08372 (9)	0.69081 (6)	0.0265 (3)	
H60	−0.0769	0.0519	0.7149	0.032*	
C61	−0.09077 (15)	0.09338 (8)	0.64126 (6)	0.0225 (3)	
H61	−0.1857	0.0685	0.6312	0.027*	
C62	−0.01504 (14)	0.13983 (8)	0.60623 (6)	0.0193 (3)	
H62	−0.0609	0.1455	0.5725	0.023*	
C63	0.15508 (12)	0.19151 (7)	0.51470 (5)	0.0147 (2)	
C64	0.13326 (13)	0.10780 (7)	0.50289 (5)	0.0164 (2)	
H64	0.1457	0.0752	0.5312	0.020*	
C65	0.09449 (13)	0.07054 (8)	0.45201 (6)	0.0189 (3)	
H65	0.0832	0.0140	0.4462	0.023*	
C66	0.07204 (14)	0.11584 (8)	0.40941 (6)	0.0203 (3)	
H66	0.0460	0.0909	0.3744	0.024*	
C67	0.08858 (14)	0.19838 (8)	0.41920 (6)	0.0201 (3)	
H67	0.0722	0.2301	0.3908	0.024*	
C68	0.12925 (13)	0.23501 (8)	0.47066 (5)	0.0174 (2)	
H68	0.1399	0.2915	0.4762	0.021*	
C69	0.18314 (14)	0.32439 (7)	0.57628 (5)	0.0178 (2)	
C70	0.05760 (15)	0.33885 (8)	0.58577 (6)	0.0231 (3)	
H70	−0.0087	0.2968	0.5981	0.028*	
C71	0.02615 (18)	0.41240 (10)	0.57785 (7)	0.0334 (4)	
H71	−0.0606	0.4194	0.5845	0.040*	
C72	0.1206 (2)	0.47546 (10)	0.56037 (7)	0.0386 (4)	
H72	0.0993	0.5256	0.5546	0.046*	
C73	0.2465 (2)	0.46410 (9)	0.55142 (7)	0.0358 (4)	
H73	0.3130	0.5069	0.5399	0.043*	
C74	0.27607 (16)	0.38996 (8)	0.55925 (6)	0.0262 (3)	
H74	0.3633	0.3836	0.5527	0.031*	
C75	0.37407 (13)	0.23679 (7)	0.59177 (5)	0.0170 (2)	
C76	0.46187 (15)	0.29513 (9)	0.62896 (6)	0.0255 (3)	
H76	0.4289	0.3385	0.6420	0.031*	
C77	0.59527 (16)	0.29242 (10)	0.64763 (7)	0.0310 (3)	
H77	0.6512	0.3338	0.6726	0.037*	
C78	0.64713 (15)	0.22949 (9)	0.62999 (6)	0.0265 (3)	
H78	0.7377	0.2271	0.6429	0.032*	
C79	0.56393 (14)	0.17049 (8)	0.59322 (6)	0.0207 (3)	
H79	0.5970	0.1267	0.5810	0.025*	
C80	0.43166 (13)	0.17531 (7)	0.57407 (5)	0.0165 (2)	
H80	0.3780	0.1352	0.5479	0.020*	
C81	0.7888 (2)	0.63258 (11)	0.27193 (8)	0.0423 (4)	
H81A	0.6998	0.6292	0.2510	0.063*	
H81B	0.7945	0.6623	0.3062	0.063*	
H81C	0.7985	0.5780	0.2777	0.063*	
C82	0.90102 (18)	0.67547 (9)	0.24293 (7)	0.0303 (3)	
C83	1.04117 (19)	0.70107 (12)	0.27313 (8)	0.0429 (4)	
H83A	1.1091	0.7130	0.2484	0.064*	
H83B	1.0579	0.6575	0.2945	0.064*	
H83C	1.0490	0.7495	0.2964	0.064*	
C84	0.5345 (2)	0.44214 (14)	0.30441 (8)	0.0484 (5)	
H84A	0.5679	0.3932	0.3032	0.073*	
H84B	0.4497	0.4315	0.3206	0.073*	
H84C	0.6042	0.4859	0.3255	0.073*	
C85	0.50690 (16)	0.46621 (10)	0.24968 (7)	0.0301 (3)	
C86	0.42688 (18)	0.52871 (11)	0.24327 (8)	0.0384 (4)	
H86A	0.4299	0.5478	0.2077	0.058*	
H86B	0.4671	0.5743	0.2697	0.058*	
H86C	0.3313	0.5050	0.2482	0.058*	
C87	0.7971 (3)	0.10881 (17)	0.02143 (12)	0.0735 (8)	
H87A	0.8724	0.1227	0.0509	0.110*	
H87B	0.8046	0.0616	0.0000	0.110*	
H87C	0.7091	0.0965	0.0354	0.110*	
C88	0.8052 (2)	0.17801 (12)	−0.01189 (9)	0.0450 (5)	
C89	0.7376 (2)	0.16136 (14)	−0.06804 (9)	0.0539 (6)	
H89A	0.7317	0.2123	−0.0829	0.081*	
H89B	0.6450	0.1260	−0.0692	0.081*	
H89C	0.7917	0.1348	−0.0888	0.081*	
C90	1.5688 (4)	0.7675 (2)	0.17338 (18)	0.1143 (14)	
H90A	1.6321	0.7445	0.1960	0.171*	
H90B	1.6082	0.8257	0.1726	0.171*	
H90C	1.5539	0.7415	0.1374	0.171*	
C91	1.4373 (2)	0.75380 (12)	0.19444 (10)	0.0533 (6)	
C92	1.4179 (4)	0.82311 (16)	0.22727 (13)	0.0810 (9)	
H92A	1.3207	0.8141	0.2316	0.121*	
H92B	1.4472	0.8730	0.2097	0.121*	
H92C	1.4729	0.8279	0.2621	0.121*	
Br1	1.2806 (2)	0.49635 (14)	0.39366 (9)	0.0274 (7)	0.1229 (17)

### Atomic displacement parameters (Å^2^)

**Table:**

	*U*^11^	*U*^22^	*U*^33^	*U*^12^	*U*^13^	*U*^23^
Co1	0.01090 (8)	0.01514 (7)	0.01354 (9)	0.00275 (6)	0.00258 (6)	0.00494 (6)
Co2	0.01629 (9)	0.01909 (8)	0.01086 (9)	0.00354 (6)	0.00324 (6)	0.00316 (6)
Cl1	0.01463 (14)	0.01972 (13)	0.02121 (16)	0.00177 (11)	0.00450 (12)	0.00756 (11)
Cl2	0.02111 (16)	0.03513 (17)	0.01254 (15)	0.00387 (13)	0.00314 (12)	0.00303 (12)
N1	0.0138 (5)	0.0186 (5)	0.0157 (5)	0.0043 (4)	0.0025 (4)	0.0045 (4)
N2	0.0155 (5)	0.0211 (5)	0.0154 (5)	0.0048 (4)	0.0015 (4)	0.0039 (4)
N3	0.0130 (5)	0.0194 (5)	0.0166 (6)	0.0033 (4)	0.0030 (4)	0.0036 (4)
N4	0.0141 (5)	0.0209 (5)	0.0176 (6)	0.0061 (4)	0.0034 (4)	0.0063 (4)
N5	0.0186 (5)	0.0240 (5)	0.0129 (5)	0.0038 (4)	0.0030 (4)	0.0030 (4)
N6	0.0246 (6)	0.0201 (5)	0.0181 (6)	0.0051 (4)	0.0054 (5)	0.0019 (4)
N7	0.0196 (6)	0.0270 (6)	0.0173 (6)	0.0036 (4)	0.0045 (5)	0.0020 (4)
N8	0.0244 (6)	0.0219 (5)	0.0159 (6)	0.0071 (4)	0.0049 (5)	0.0052 (4)
B1	0.0170 (7)	0.0165 (6)	0.0175 (7)	0.0038 (5)	0.0016 (5)	0.0006 (5)
B2	0.0143 (6)	0.0161 (6)	0.0166 (7)	0.0041 (5)	0.0026 (5)	0.0007 (5)
O1	0.0724 (10)	0.0352 (6)	0.0257 (7)	0.0093 (6)	−0.0001 (6)	−0.0037 (5)
O2	0.0429 (7)	0.0486 (7)	0.0382 (7)	0.0023 (6)	0.0244 (6)	−0.0037 (6)
O3	0.0676 (11)	0.0562 (9)	0.0624 (11)	0.0088 (8)	0.0078 (8)	−0.0306 (8)
O4	0.0910 (15)	0.0516 (11)	0.139 (2)	−0.0102 (10)	−0.0482 (14)	0.0027 (12)
C1	0.0255 (13)	0.0393 (12)	0.0347 (14)	0.0008 (10)	−0.0039 (11)	0.0006 (9)
C2	0.0237 (16)	0.0235 (12)	0.0203 (15)	0.0048 (10)	0.0033 (10)	0.0035 (9)
C3	0.0169 (6)	0.0161 (5)	0.0172 (6)	0.0039 (4)	0.0029 (5)	0.0002 (4)
C4	0.0184 (6)	0.0175 (5)	0.0149 (6)	0.0055 (5)	0.0037 (5)	0.0037 (4)
C5	0.0170 (6)	0.0158 (5)	0.0157 (6)	0.0051 (4)	0.0064 (5)	0.0031 (4)
C6	0.0156 (6)	0.0177 (5)	0.0154 (6)	0.0031 (4)	0.0041 (5)	0.0027 (4)
C7	0.0194 (6)	0.0160 (5)	0.0130 (6)	0.0053 (4)	0.0054 (5)	0.0025 (4)
C8	0.0183 (6)	0.0162 (5)	0.0163 (6)	0.0024 (4)	0.0057 (5)	0.0027 (4)
C9	0.0188 (6)	0.0200 (6)	0.0154 (6)	0.0061 (5)	0.0036 (5)	0.0042 (5)
C10	0.0171 (6)	0.0197 (6)	0.0164 (6)	0.0070 (5)	0.0016 (5)	0.0024 (5)
C11	0.0200 (6)	0.0181 (5)	0.0185 (7)	0.0037 (5)	0.0055 (5)	0.0037 (5)
C12	0.0175 (6)	0.0182 (5)	0.0183 (7)	0.0032 (5)	0.0037 (5)	0.0035 (5)
C13	0.0266 (7)	0.0259 (7)	0.0260 (8)	0.0124 (6)	0.0005 (6)	−0.0034 (6)
C14	0.0255 (7)	0.0220 (6)	0.0227 (7)	0.0076 (5)	−0.0022 (6)	−0.0032 (5)
C15	0.0171 (6)	0.0283 (7)	0.0193 (7)	0.0065 (5)	−0.0013 (5)	0.0028 (5)
C16	0.0204 (6)	0.0295 (7)	0.0189 (7)	0.0104 (5)	−0.0001 (5)	0.0072 (5)
C17	0.0219 (7)	0.0189 (6)	0.0254 (7)	0.0087 (5)	0.0077 (5)	0.0075 (5)
C18	0.0230 (7)	0.0192 (6)	0.0245 (7)	0.0071 (5)	0.0084 (6)	0.0028 (5)
C19	0.0207 (6)	0.0197 (6)	0.0223 (7)	0.0032 (5)	0.0046 (5)	0.0000 (5)
C20	0.0145 (6)	0.0277 (6)	0.0194 (7)	0.0050 (5)	−0.0001 (5)	0.0001 (5)
C21	0.0159 (6)	0.0275 (6)	0.0163 (6)	0.0079 (5)	0.0009 (5)	0.0044 (5)
C22	0.0201 (6)	0.0184 (6)	0.0251 (7)	0.0082 (5)	0.0005 (5)	0.0035 (5)
C23	0.0308 (8)	0.0219 (6)	0.0227 (7)	0.0030 (6)	0.0085 (6)	0.0062 (5)
C24	0.0240 (7)	0.0240 (6)	0.0214 (7)	−0.0010 (5)	0.0042 (6)	0.0000 (5)
C25	0.0173 (6)	0.0368 (8)	0.0188 (7)	0.0079 (6)	0.0023 (5)	0.0067 (6)
C26	0.0270 (7)	0.0368 (8)	0.0248 (8)	0.0171 (6)	0.0062 (6)	0.0063 (6)
C27	0.0319 (8)	0.0252 (7)	0.0251 (8)	0.0134 (6)	0.0066 (6)	0.0064 (6)
C28	0.0310 (8)	0.0207 (6)	0.0292 (8)	−0.0008 (6)	0.0098 (6)	0.0029 (6)
C29	0.0236 (7)	0.0277 (7)	0.0273 (8)	−0.0043 (6)	0.0067 (6)	−0.0020 (6)
C30	0.0181 (7)	0.0452 (9)	0.0260 (8)	0.0088 (6)	0.0038 (6)	0.0062 (7)
C31	0.0267 (8)	0.0452 (9)	0.0327 (9)	0.0189 (7)	0.0088 (7)	0.0099 (7)
C32	0.0324 (8)	0.0326 (7)	0.0284 (8)	0.0173 (6)	0.0054 (6)	0.0112 (6)
C33	0.0195 (6)	0.0177 (5)	0.0209 (7)	0.0045 (5)	0.0050 (5)	−0.0013 (5)
C34	0.0273 (7)	0.0211 (6)	0.0216 (7)	0.0040 (5)	0.0069 (6)	0.0005 (5)
C35	0.0352 (8)	0.0213 (6)	0.0314 (9)	0.0016 (6)	0.0142 (7)	0.0021 (6)
C36	0.0281 (8)	0.0233 (7)	0.0477 (11)	−0.0052 (6)	0.0079 (7)	−0.0034 (7)
C37	0.0286 (8)	0.0302 (8)	0.0425 (10)	−0.0026 (6)	−0.0082 (7)	−0.0029 (7)
C38	0.0256 (7)	0.0227 (6)	0.0301 (8)	0.0011 (5)	−0.0035 (6)	0.0007 (6)
C39	0.0167 (6)	0.0199 (6)	0.0176 (6)	0.0056 (5)	−0.0014 (5)	−0.0001 (5)
C40	0.0157 (6)	0.0238 (6)	0.0232 (7)	0.0040 (5)	0.0001 (5)	−0.0023 (5)
C41	0.0160 (6)	0.0348 (7)	0.0246 (8)	0.0072 (5)	0.0020 (5)	−0.0030 (6)
C42	0.0249 (7)	0.0326 (7)	0.0227 (7)	0.0162 (6)	−0.0043 (6)	−0.0069 (6)
C43	0.0311 (8)	0.0205 (6)	0.0253 (8)	0.0095 (6)	−0.0029 (6)	−0.0031 (5)
C44	0.0244 (7)	0.0198 (6)	0.0221 (7)	0.0061 (5)	0.0024 (6)	0.0011 (5)
C45	0.0194 (6)	0.0199 (6)	0.0149 (6)	0.0044 (5)	0.0026 (5)	0.0031 (5)
C46	0.0217 (7)	0.0217 (6)	0.0181 (7)	0.0042 (5)	0.0033 (5)	0.0023 (5)
C47	0.0205 (7)	0.0297 (7)	0.0252 (8)	0.0011 (5)	−0.0007 (6)	0.0022 (6)
C48	0.0200 (7)	0.0375 (8)	0.0361 (9)	0.0090 (6)	−0.0001 (6)	0.0048 (7)
C49	0.0275 (8)	0.0294 (7)	0.0393 (10)	0.0141 (6)	0.0024 (7)	0.0010 (6)
C50	0.0232 (7)	0.0219 (6)	0.0275 (8)	0.0066 (5)	−0.0007 (6)	−0.0012 (5)
C51	0.0177 (6)	0.0165 (5)	0.0181 (7)	0.0013 (5)	−0.0006 (5)	−0.0014 (5)
C52	0.0247 (7)	0.0247 (6)	0.0228 (7)	0.0091 (5)	0.0020 (6)	0.0019 (5)
C53	0.0370 (9)	0.0279 (7)	0.0241 (8)	0.0125 (6)	−0.0021 (7)	0.0044 (6)
C54	0.0357 (9)	0.0307 (7)	0.0183 (7)	0.0049 (6)	0.0013 (6)	0.0048 (6)
C55	0.0299 (8)	0.0411 (8)	0.0223 (8)	0.0111 (7)	0.0088 (6)	0.0033 (6)
C56	0.0258 (7)	0.0292 (7)	0.0223 (7)	0.0108 (6)	0.0041 (6)	0.0037 (6)
C57	0.0173 (6)	0.0154 (5)	0.0163 (6)	0.0056 (4)	0.0030 (5)	0.0001 (4)
C58	0.0202 (6)	0.0267 (6)	0.0185 (7)	0.0067 (5)	0.0017 (5)	0.0017 (5)
C59	0.0321 (8)	0.0337 (7)	0.0174 (7)	0.0087 (6)	0.0033 (6)	0.0060 (6)
C60	0.0329 (8)	0.0258 (7)	0.0212 (7)	0.0042 (6)	0.0111 (6)	0.0047 (5)
C61	0.0214 (7)	0.0220 (6)	0.0221 (7)	0.0007 (5)	0.0070 (5)	−0.0014 (5)
C62	0.0189 (6)	0.0214 (6)	0.0168 (7)	0.0036 (5)	0.0025 (5)	0.0014 (5)
C63	0.0111 (5)	0.0160 (5)	0.0175 (6)	0.0037 (4)	0.0034 (5)	0.0015 (4)
C64	0.0129 (6)	0.0177 (5)	0.0190 (6)	0.0041 (4)	0.0028 (5)	0.0024 (5)
C65	0.0143 (6)	0.0192 (6)	0.0232 (7)	0.0038 (5)	0.0043 (5)	−0.0021 (5)
C66	0.0153 (6)	0.0275 (6)	0.0174 (7)	0.0042 (5)	0.0035 (5)	−0.0021 (5)
C67	0.0169 (6)	0.0268 (6)	0.0175 (7)	0.0063 (5)	0.0031 (5)	0.0054 (5)
C68	0.0161 (6)	0.0177 (5)	0.0192 (7)	0.0052 (4)	0.0036 (5)	0.0027 (5)
C69	0.0213 (6)	0.0168 (5)	0.0155 (6)	0.0056 (5)	0.0025 (5)	−0.0013 (4)
C70	0.0246 (7)	0.0222 (6)	0.0236 (7)	0.0082 (5)	0.0045 (6)	−0.0014 (5)
C71	0.0366 (9)	0.0311 (8)	0.0389 (10)	0.0197 (7)	0.0084 (7)	−0.0014 (7)
C72	0.0589 (12)	0.0230 (7)	0.0418 (10)	0.0226 (7)	0.0122 (9)	0.0040 (7)
C73	0.0510 (11)	0.0175 (6)	0.0427 (10)	0.0089 (7)	0.0200 (8)	0.0051 (6)
C74	0.0301 (8)	0.0190 (6)	0.0321 (8)	0.0066 (5)	0.0134 (6)	0.0014 (5)
C75	0.0154 (6)	0.0174 (5)	0.0175 (6)	0.0024 (4)	0.0034 (5)	0.0023 (5)
C76	0.0200 (7)	0.0256 (7)	0.0289 (8)	0.0050 (5)	0.0001 (6)	−0.0071 (6)
C77	0.0208 (7)	0.0339 (8)	0.0325 (9)	0.0025 (6)	−0.0055 (6)	−0.0099 (6)
C78	0.0159 (6)	0.0346 (7)	0.0277 (8)	0.0061 (6)	−0.0014 (6)	0.0014 (6)
C79	0.0191 (6)	0.0230 (6)	0.0222 (7)	0.0073 (5)	0.0056 (5)	0.0057 (5)
C80	0.0155 (6)	0.0178 (5)	0.0155 (6)	0.0025 (4)	0.0029 (5)	0.0034 (4)
C81	0.0384 (10)	0.0387 (9)	0.0443 (11)	−0.0006 (8)	0.0092 (8)	−0.0065 (8)
C82	0.0371 (9)	0.0226 (7)	0.0287 (9)	0.0052 (6)	0.0030 (7)	−0.0070 (6)
C83	0.0327 (9)	0.0412 (10)	0.0517 (12)	0.0068 (8)	0.0011 (8)	−0.0010 (8)
C84	0.0492 (12)	0.0715 (14)	0.0376 (11)	0.0296 (10)	0.0210 (9)	0.0222 (10)
C85	0.0248 (7)	0.0333 (8)	0.0317 (9)	0.0023 (6)	0.0121 (6)	0.0036 (6)
C86	0.0303 (9)	0.0391 (9)	0.0453 (11)	0.0075 (7)	0.0058 (8)	0.0050 (8)
C87	0.0743 (18)	0.0729 (17)	0.082 (2)	0.0328 (14)	0.0154 (15)	0.0105 (15)
C88	0.0359 (10)	0.0458 (10)	0.0536 (12)	0.0137 (8)	0.0084 (9)	−0.0158 (9)
C89	0.0457 (12)	0.0625 (13)	0.0488 (13)	0.0077 (10)	0.0092 (10)	−0.0198 (10)
C90	0.110 (3)	0.072 (2)	0.180 (4)	0.039 (2)	0.065 (3)	−0.002 (2)
C91	0.0527 (13)	0.0383 (10)	0.0602 (14)	0.0082 (9)	−0.0199 (11)	0.0069 (9)
C92	0.109 (2)	0.0525 (14)	0.089 (2)	0.0184 (15)	0.0442 (19)	0.0199 (14)
Br1	0.0193 (17)	0.0347 (12)	0.0254 (14)	0.0051 (11)	−0.0031 (10)	−0.0002 (8)

### Geometric parameters (Å, °)

**Table:**

Co1—C10	1.8783 (13)	C34—C35	1.395 (2)
Co1—N3	1.9724 (11)	C34—H34	0.9500
Co1—N4	1.9755 (11)	C35—C36	1.384 (2)
Co1—N1	1.9792 (10)	C35—H35	0.9500
Co1—N2	1.9793 (11)	C36—C37	1.386 (3)
Co1—Cl1	2.2988 (3)	C36—H36	0.9500
Co2—C12	1.8756 (14)	C37—C38	1.396 (2)
Co2—N5	1.9700 (11)	C37—H37	0.9500
Co2—N8	1.9737 (11)	C38—H38	0.9500
Co2—N6	1.9777 (11)	C39—C40	1.406 (2)
Co2—N7	1.9858 (12)	C39—C44	1.4116 (18)
Co2—Cl2	2.3164 (4)	C40—C41	1.394 (2)
N1—C22	1.4835 (17)	C40—H40	0.9500
N1—C21	1.4855 (17)	C41—C42	1.385 (2)
N1—H1	0.9300	C41—H41	0.9500
N2—C15	1.4847 (17)	C42—C43	1.379 (2)
N2—C14	1.4861 (17)	C42—H42	0.9500
N2—H2	0.9300	C43—C44	1.393 (2)
N3—C19	1.4843 (17)	C43—H43	0.9500
N3—C20	1.4849 (17)	C44—H44	0.9500
N3—H3	0.9300	C45—C50	1.4048 (19)
N4—C16	1.4791 (18)	C45—C46	1.4061 (19)
N4—C17	1.4860 (17)	C46—C47	1.398 (2)
N4—H4	0.9300	C46—H46	0.9500
N5—C25	1.4842 (17)	C47—C48	1.388 (2)
N5—C24	1.4877 (18)	C47—H47	0.9500
N5—H5	0.9300	C48—C49	1.384 (2)
N6—C27	1.4827 (19)	C48—H48	0.9500
N6—C28	1.4931 (18)	C49—C50	1.393 (2)
N6—H6	0.9300	C49—H49	0.9500
N7—C30	1.4825 (19)	C50—H50	0.9500
N7—C29	1.4861 (19)	C51—C56	1.404 (2)
N7—H7	0.9300	C51—C52	1.4049 (19)
N8—C32	1.4848 (19)	C52—C53	1.392 (2)
N8—C23	1.4884 (18)	C52—H52	0.9500
N8—H8	0.9300	C53—C54	1.382 (2)
B1—C33	1.6425 (19)	C53—H53	0.9500
B1—C39	1.652 (2)	C54—C55	1.379 (2)
B1—C51	1.652 (2)	C54—H54	0.9500
B1—C45	1.653 (2)	C55—C56	1.386 (2)
B2—C69	1.6431 (18)	C55—H55	0.9500
B2—C75	1.6503 (19)	C56—H56	0.9500
B2—C63	1.654 (2)	C57—C58	1.4028 (19)
B2—C57	1.656 (2)	C57—C62	1.4044 (18)
O1—C82	1.216 (2)	C58—C59	1.395 (2)
O2—C85	1.210 (2)	C58—H58	0.9500
O3—C88	1.209 (2)	C59—C60	1.385 (2)
O4—C91	1.198 (3)	C59—H59	0.9500
C1—C2	1.192 (5)	C60—C61	1.387 (2)
C1—H1A	0.9500	C60—H60	0.9500
C2—C3	1.426 (3)	C61—C62	1.3963 (19)
C3—C4	1.3943 (18)	C61—H61	0.9500
C3—C8	1.3973 (18)	C62—H62	0.9500
C3—Br1	1.947 (3)	C63—C64	1.4095 (17)
C4—C5	1.4013 (18)	C63—C68	1.4109 (18)
C4—H4A	0.9500	C64—C65	1.3883 (19)
C5—C6	1.3960 (18)	C64—H64	0.9500
C5—C9	1.4392 (18)	C65—C66	1.394 (2)
C6—C7	1.4000 (17)	C65—H65	0.9500
C6—H6A	0.9500	C66—C67	1.3903 (19)
C7—C8	1.3978 (18)	C66—H66	0.9500
C7—C11	1.4384 (18)	C67—C68	1.3987 (19)
C8—H8A	0.9500	C67—H67	0.9500
C9—C10	1.2027 (18)	C68—H68	0.9500
C11—C12	1.2007 (19)	C69—C74	1.3997 (19)
C13—C22	1.512 (2)	C69—C70	1.4022 (19)
C13—C14	1.517 (2)	C70—C71	1.392 (2)
C13—H13A	0.9900	C70—H70	0.9500
C13—H13B	0.9900	C71—C72	1.386 (3)
C14—H14A	0.9900	C71—H71	0.9500
C14—H14B	0.9900	C72—C73	1.381 (3)
C15—C16	1.508 (2)	C72—H72	0.9500
C15—H15A	0.9900	C73—C74	1.394 (2)
C15—H15B	0.9900	C73—H73	0.9500
C16—H16A	0.9900	C74—H74	0.9500
C16—H16B	0.9900	C75—C76	1.4019 (19)
C17—C18	1.514 (2)	C75—C80	1.4072 (17)
C17—H17A	0.9900	C76—C77	1.393 (2)
C17—H17B	0.9900	C76—H76	0.9500
C18—C19	1.5197 (19)	C77—C78	1.391 (2)
C18—H18A	0.9900	C77—H77	0.9500
C18—H18B	0.9900	C78—C79	1.385 (2)
C19—H19A	0.9900	C78—H78	0.9500
C19—H19B	0.9900	C79—C80	1.3956 (18)
C20—C21	1.5094 (19)	C79—H79	0.9500
C20—H20A	0.9900	C80—H80	0.9500
C20—H20B	0.9900	C81—C82	1.490 (3)
C21—H21A	0.9900	C81—H81A	0.9800
C21—H21B	0.9900	C81—H81B	0.9800
C22—H22A	0.9900	C81—H81C	0.9800
C22—H22B	0.9900	C82—C83	1.485 (2)
C23—C24	1.502 (2)	C83—H83A	0.9800
C23—H23A	0.9900	C83—H83B	0.9800
C23—H23B	0.9900	C83—H83C	0.9800
C24—H24A	0.9900	C84—C85	1.489 (3)
C24—H24B	0.9900	C84—H84A	0.9800
C25—C26	1.515 (2)	C84—H84B	0.9800
C25—H25A	0.9900	C84—H84C	0.9800
C25—H25B	0.9900	C85—C86	1.499 (2)
C26—C27	1.515 (2)	C86—H86A	0.9800
C26—H26A	0.9900	C86—H86B	0.9800
C26—H26B	0.9900	C86—H86C	0.9800
C27—H27A	0.9900	C87—C88	1.482 (4)
C27—H27B	0.9900	C87—H87A	0.9800
C28—C29	1.505 (2)	C87—H87B	0.9800
C28—H28A	0.9900	C87—H87C	0.9800
C28—H28B	0.9900	C88—C89	1.496 (3)
C29—H29A	0.9900	C89—H89A	0.9800
C29—H29B	0.9900	C89—H89B	0.9800
C30—C31	1.514 (2)	C89—H89C	0.9800
C30—H30A	0.9900	C90—C91	1.466 (4)
C30—H30B	0.9900	C90—H90A	0.9800
C31—C32	1.518 (2)	C90—H90B	0.9800
C31—H31A	0.9900	C90—H90C	0.9800
C31—H31B	0.9900	C91—C92	1.489 (4)
C32—H32A	0.9900	C92—H92A	0.9800
C32—H32B	0.9900	C92—H92B	0.9800
C33—C38	1.398 (2)	C92—H92C	0.9800
C33—C34	1.4053 (19)		
			
C10—Co1—N3	90.39 (5)	C31—C30—H30A	109.2
C10—Co1—N4	92.33 (5)	N7—C30—H30B	109.2
N3—Co1—N4	92.75 (5)	C31—C30—H30B	109.2
C10—Co1—N1	87.66 (5)	H30A—C30—H30B	107.9
N3—Co1—N1	86.53 (4)	C30—C31—C32	113.79 (13)
N4—Co1—N1	179.27 (5)	C30—C31—H31A	108.8
C10—Co1—N2	89.89 (5)	C32—C31—H31A	108.8
N3—Co1—N2	178.99 (4)	C30—C31—H31B	108.8
N4—Co1—N2	86.27 (5)	C32—C31—H31B	108.8
N1—Co1—N2	94.45 (5)	H31A—C31—H31B	107.7
C10—Co1—Cl1	178.32 (4)	N8—C32—C31	111.89 (12)
N3—Co1—Cl1	88.24 (3)	N8—C32—H32A	109.2
N4—Co1—Cl1	88.70 (3)	C31—C32—H32A	109.2
N1—Co1—Cl1	91.29 (3)	N8—C32—H32B	109.2
N2—Co1—Cl1	91.49 (3)	C31—C32—H32B	109.2
C12—Co2—N5	89.92 (5)	H32A—C32—H32B	107.9
C12—Co2—N8	90.84 (5)	C38—C33—C34	115.02 (13)
N5—Co2—N8	86.22 (5)	C38—C33—B1	122.53 (12)
C12—Co2—N6	89.17 (5)	C34—C33—B1	122.23 (12)
N5—Co2—N6	93.72 (5)	C35—C34—C33	123.09 (15)
N8—Co2—N6	179.94 (6)	C35—C34—H34	118.5
C12—Co2—N7	89.23 (5)	C33—C34—H34	118.5
N5—Co2—N7	179.14 (5)	C36—C35—C34	119.77 (15)
N8—Co2—N7	93.66 (5)	C36—C35—H35	120.1
N6—Co2—N7	86.40 (5)	C34—C35—H35	120.1
C12—Co2—Cl2	177.48 (4)	C35—C36—C37	119.17 (14)
N5—Co2—Cl2	89.35 (4)	C35—C36—H36	120.4
N8—Co2—Cl2	91.52 (4)	C37—C36—H36	120.4
N6—Co2—Cl2	88.47 (4)	C36—C37—C38	120.08 (16)
N7—Co2—Cl2	91.50 (4)	C36—C37—H37	120.0
C22—N1—C21	111.44 (10)	C38—C37—H37	120.0
C22—N1—Co1	118.63 (8)	C37—C38—C33	122.86 (15)
C21—N1—Co1	107.18 (8)	C37—C38—H38	118.6
C22—N1—H1	106.3	C33—C38—H38	118.6
C21—N1—H1	106.3	C40—C39—C44	114.61 (12)
Co1—N1—H1	106.3	C40—C39—B1	123.29 (11)
C15—N2—C14	111.18 (11)	C44—C39—B1	122.10 (12)
C15—N2—Co1	107.35 (8)	C41—C40—C39	122.97 (13)
C14—N2—Co1	119.67 (9)	C41—C40—H40	118.5
C15—N2—H2	105.9	C39—C40—H40	118.5
C14—N2—H2	105.9	C42—C41—C40	120.40 (14)
Co1—N2—H2	105.9	C42—C41—H41	119.8
C19—N3—C20	110.57 (10)	C40—C41—H41	119.8
C19—N3—Co1	120.30 (9)	C43—C42—C41	118.57 (13)
C20—N3—Co1	107.98 (8)	C43—C42—H42	120.7
C19—N3—H3	105.6	C41—C42—H42	120.7
C20—N3—H3	105.6	C42—C43—C44	120.81 (13)
Co1—N3—H3	105.6	C42—C43—H43	119.6
C16—N4—C17	110.99 (10)	C44—C43—H43	119.6
C16—N4—Co1	107.97 (8)	C43—C44—C39	122.63 (14)
C17—N4—Co1	118.43 (8)	C43—C44—H44	118.7
C16—N4—H4	106.2	C39—C44—H44	118.7
C17—N4—H4	106.2	C50—C45—C46	115.03 (12)
Co1—N4—H4	106.2	C50—C45—B1	120.59 (12)
C25—N5—C24	111.17 (11)	C46—C45—B1	123.65 (11)
C25—N5—Co2	119.50 (9)	C47—C46—C45	122.58 (13)
C24—N5—Co2	108.19 (9)	C47—C46—H46	118.7
C25—N5—H5	105.7	C45—C46—H46	118.7
C24—N5—H5	105.7	C48—C47—C46	120.18 (14)
Co2—N5—H5	105.7	C48—C47—H47	119.9
C27—N6—C28	110.89 (11)	C46—C47—H47	119.9
C27—N6—Co2	119.69 (9)	C49—C48—C47	119.04 (14)
C28—N6—Co2	107.78 (9)	C49—C48—H48	120.5
C27—N6—H6	105.8	C47—C48—H48	120.5
C28—N6—H6	105.8	C48—C49—C50	120.04 (14)
Co2—N6—H6	105.8	C48—C49—H49	120.0
C30—N7—C29	111.23 (12)	C50—C49—H49	120.0
C30—N7—Co2	118.84 (9)	C49—C50—C45	123.10 (14)
C29—N7—Co2	107.59 (9)	C49—C50—H50	118.5
C30—N7—H7	106.1	C45—C50—H50	118.5
C29—N7—H7	106.1	C56—C51—C52	114.81 (13)
Co2—N7—H7	106.1	C56—C51—B1	122.44 (12)
C32—N8—C23	111.27 (11)	C52—C51—B1	122.65 (12)
C32—N8—Co2	119.11 (10)	C53—C52—C51	122.80 (14)
C23—N8—Co2	107.87 (9)	C53—C52—H52	118.6
C32—N8—H8	105.9	C51—C52—H52	118.6
C23—N8—H8	105.9	C54—C53—C52	120.21 (14)
Co2—N8—H8	105.9	C54—C53—H53	119.9
C33—B1—C39	108.24 (11)	C52—C53—H53	119.9
C33—B1—C51	109.11 (11)	C55—C54—C53	118.82 (14)
C39—B1—C51	110.31 (10)	C55—C54—H54	120.6
C33—B1—C45	111.16 (10)	C53—C54—H54	120.6
C39—B1—C45	112.18 (11)	C54—C55—C56	120.55 (15)
C51—B1—C45	105.80 (11)	C54—C55—H55	119.7
C69—B2—C75	112.19 (10)	C56—C55—H55	119.7
C69—B2—C63	106.59 (10)	C55—C56—C51	122.82 (14)
C75—B2—C63	110.50 (10)	C55—C56—H56	118.6
C69—B2—C57	111.05 (10)	C51—C56—H56	118.6
C75—B2—C57	106.73 (10)	C58—C57—C62	114.62 (12)
C63—B2—C57	109.81 (10)	C58—C57—B2	123.68 (12)
C2—C1—H1A	180.0	C62—C57—B2	121.70 (11)
C1—C2—C3	179.3 (4)	C59—C58—C57	122.79 (14)
C4—C3—C8	120.41 (12)	C59—C58—H58	118.6
C4—C3—C2	119.67 (17)	C57—C58—H58	118.6
C8—C3—C2	119.91 (17)	C60—C59—C58	120.61 (14)
C4—C3—Br1	120.24 (12)	C60—C59—H59	119.7
C8—C3—Br1	119.31 (12)	C58—C59—H59	119.7
C3—C4—C5	120.10 (12)	C59—C60—C61	118.73 (14)
C3—C4—H4A	120.0	C59—C60—H60	120.6
C5—C4—H4A	120.0	C61—C60—H60	120.6
C6—C5—C4	119.17 (11)	C60—C61—C62	119.72 (13)
C6—C5—C9	119.98 (12)	C60—C61—H61	120.1
C4—C5—C9	120.85 (12)	C62—C61—H61	120.1
C5—C6—C7	121.03 (12)	C61—C62—C57	123.53 (13)
C5—C6—H6A	119.5	C61—C62—H62	118.2
C7—C6—H6A	119.5	C57—C62—H62	118.2
C8—C7—C6	119.32 (12)	C64—C63—C68	114.59 (12)
C8—C7—C11	122.29 (12)	C64—C63—B2	121.56 (11)
C6—C7—C11	118.26 (12)	C68—C63—B2	123.83 (11)
C3—C8—C7	119.94 (12)	C65—C64—C63	123.44 (12)
C3—C8—H8A	120.0	C65—C64—H64	118.3
C7—C8—H8A	120.0	C63—C64—H64	118.3
C10—C9—C5	178.08 (14)	C64—C65—C66	120.20 (12)
C9—C10—Co1	173.79 (12)	C64—C65—H65	119.9
C12—C11—C7	169.37 (14)	C66—C65—H65	119.9
C11—C12—Co2	171.04 (12)	C67—C66—C65	118.55 (13)
C22—C13—C14	113.56 (12)	C67—C66—H66	120.7
C22—C13—H13A	108.9	C65—C66—H66	120.7
C14—C13—H13A	108.9	C66—C67—C68	120.43 (12)
C22—C13—H13B	108.9	C66—C67—H67	119.8
C14—C13—H13B	108.9	C68—C67—H67	119.8
H13A—C13—H13B	107.7	C67—C68—C63	122.75 (12)
N2—C14—C13	111.62 (11)	C67—C68—H68	118.6
N2—C14—H14A	109.3	C63—C68—H68	118.6
C13—C14—H14A	109.3	C74—C69—C70	115.08 (12)
N2—C14—H14B	109.3	C74—C69—B2	121.57 (12)
C13—C14—H14B	109.3	C70—C69—B2	122.61 (12)
H14A—C14—H14B	108.0	C71—C70—C69	122.65 (14)
N2—C15—C16	106.66 (11)	C71—C70—H70	118.7
N2—C15—H15A	110.4	C69—C70—H70	118.7
C16—C15—H15A	110.4	C72—C71—C70	120.37 (15)
N2—C15—H15B	110.4	C72—C71—H71	119.8
C16—C15—H15B	110.4	C70—C71—H71	119.8
H15A—C15—H15B	108.6	C73—C72—C71	118.77 (14)
N4—C16—C15	107.56 (11)	C73—C72—H72	120.6
N4—C16—H16A	110.2	C71—C72—H72	120.6
C15—C16—H16A	110.2	C72—C73—C74	120.15 (15)
N4—C16—H16B	110.2	C72—C73—H73	119.9
C15—C16—H16B	110.2	C74—C73—H73	119.9
H16A—C16—H16B	108.5	C73—C74—C69	122.97 (15)
N4—C17—C18	111.90 (10)	C73—C74—H74	118.5
N4—C17—H17A	109.2	C69—C74—H74	118.5
C18—C17—H17A	109.2	C76—C75—C80	114.78 (12)
N4—C17—H17B	109.2	C76—C75—B2	122.69 (12)
C18—C17—H17B	109.2	C80—C75—B2	122.12 (11)
H17A—C17—H17B	107.9	C77—C76—C75	122.97 (13)
C17—C18—C19	114.00 (11)	C77—C76—H76	118.5
C17—C18—H18A	108.8	C75—C76—H76	118.5
C19—C18—H18A	108.8	C78—C77—C76	120.38 (14)
C17—C18—H18B	108.8	C78—C77—H77	119.8
C19—C18—H18B	108.8	C76—C77—H77	119.8
H18A—C18—H18B	107.6	C79—C78—C77	118.62 (13)
N3—C19—C18	111.67 (11)	C79—C78—H78	120.7
N3—C19—H19A	109.3	C77—C78—H78	120.7
C18—C19—H19A	109.3	C78—C79—C80	120.13 (13)
N3—C19—H19B	109.3	C78—C79—H79	119.9
C18—C19—H19B	109.3	C80—C79—H79	119.9
H19A—C19—H19B	107.9	C79—C80—C75	123.08 (12)
N3—C20—C21	107.42 (11)	C79—C80—H80	118.5
N3—C20—H20A	110.2	C75—C80—H80	118.5
C21—C20—H20A	110.2	C82—C81—H81A	109.5
N3—C20—H20B	110.2	C82—C81—H81B	109.5
C21—C20—H20B	110.2	H81A—C81—H81B	109.5
H20A—C20—H20B	108.5	C82—C81—H81C	109.5
N1—C21—C20	106.89 (10)	H81A—C81—H81C	109.5
N1—C21—H21A	110.3	H81B—C81—H81C	109.5
C20—C21—H21A	110.3	O1—C82—C83	121.67 (17)
N1—C21—H21B	110.3	O1—C82—C81	121.55 (17)
C20—C21—H21B	110.3	C83—C82—C81	116.78 (16)
H21A—C21—H21B	108.6	C82—C83—H83A	109.5
N1—C22—C13	112.21 (11)	C82—C83—H83B	109.5
N1—C22—H22A	109.2	H83A—C83—H83B	109.5
C13—C22—H22A	109.2	C82—C83—H83C	109.5
N1—C22—H22B	109.2	H83A—C83—H83C	109.5
C13—C22—H22B	109.2	H83B—C83—H83C	109.5
H22A—C22—H22B	107.9	C85—C84—H84A	109.5
N8—C23—C24	107.16 (11)	C85—C84—H84B	109.5
N8—C23—H23A	110.3	H84A—C84—H84B	109.5
C24—C23—H23A	110.3	C85—C84—H84C	109.5
N8—C23—H23B	110.3	H84A—C84—H84C	109.5
C24—C23—H23B	110.3	H84B—C84—H84C	109.5
H23A—C23—H23B	108.5	O2—C85—C84	121.66 (17)
N5—C24—C23	107.02 (11)	O2—C85—C86	122.14 (17)
N5—C24—H24A	110.3	C84—C85—C86	116.21 (15)
C23—C24—H24A	110.3	C85—C86—H86A	109.5
N5—C24—H24B	110.3	C85—C86—H86B	109.5
C23—C24—H24B	110.3	H86A—C86—H86B	109.5
H24A—C24—H24B	108.6	C85—C86—H86C	109.5
N5—C25—C26	111.35 (12)	H86A—C86—H86C	109.5
N5—C25—H25A	109.4	H86B—C86—H86C	109.5
C26—C25—H25A	109.4	C88—C87—H87A	109.5
N5—C25—H25B	109.4	C88—C87—H87B	109.5
C26—C25—H25B	109.4	H87A—C87—H87B	109.5
H25A—C25—H25B	108.0	C88—C87—H87C	109.5
C27—C26—C25	114.01 (12)	H87A—C87—H87C	109.5
C27—C26—H26A	108.7	H87B—C87—H87C	109.5
C25—C26—H26A	108.7	O3—C88—C87	122.1 (2)
C27—C26—H26B	108.7	O3—C88—C89	119.9 (2)
C25—C26—H26B	108.7	C87—C88—C89	118.0 (2)
H26A—C26—H26B	107.6	C88—C89—H89A	109.5
N6—C27—C26	111.46 (12)	C88—C89—H89B	109.5
N6—C27—H27A	109.3	H89A—C89—H89B	109.5
C26—C27—H27A	109.3	C88—C89—H89C	109.5
N6—C27—H27B	109.3	H89A—C89—H89C	109.5
C26—C27—H27B	109.3	H89B—C89—H89C	109.5
H27A—C27—H27B	108.0	C91—C90—H90A	109.5
N6—C28—C29	107.21 (12)	C91—C90—H90B	109.5
N6—C28—H28A	110.3	H90A—C90—H90B	109.5
C29—C28—H28A	110.3	C91—C90—H90C	109.5
N6—C28—H28B	110.3	H90A—C90—H90C	109.5
C29—C28—H28B	110.3	H90B—C90—H90C	109.5
H28A—C28—H28B	108.5	O4—C91—C90	123.3 (3)
N7—C29—C28	107.56 (12)	O4—C91—C92	121.1 (3)
N7—C29—H29A	110.2	C90—C91—C92	115.5 (2)
C28—C29—H29A	110.2	C91—C92—H92A	109.5
N7—C29—H29B	110.2	C91—C92—H92B	109.5
C28—C29—H29B	110.2	H92A—C92—H92B	109.5
H29A—C29—H29B	108.5	C91—C92—H92C	109.5
N7—C30—C31	111.89 (13)	H92A—C92—H92C	109.5
N7—C30—H30A	109.2	H92B—C92—H92C	109.5

### Hydrogen-bond geometry (Å, °)

**Table:**

*D*—H···*A*	*D*—H	H···*A*	*D*···*A*	*D*—H···*A*
N5—H5···Cl2^i^	0.93	2.48	3.2377 (12)	139.
N6—H6···O3	0.93	2.15	2.9440 (19)	143.
N7—H7···O2	0.93	2.11	2.9894 (17)	157.
N8—H8···O1	0.93	2.03	2.8730 (17)	149.

Symmetry codes: (i) −*x*+2, −*y*+1, −*z*.
